# Self-Efficacy Beliefs of University Students: Examining Factor Validity and Measurement Invariance of the New Academic Self-Efficacy Scale

**DOI:** 10.3389/fpsyg.2021.498824

**Published:** 2022-01-13

**Authors:** Andrea Greco, Chiara Annovazzi, Nicola Palena, Elisabetta Camussi, Germano Rossi, Patrizia Steca

**Affiliations:** ^1^Department of Human and Social Sciences, University of Bergamo, Bergamo, Italy; ^2^Department of Psychology, University of Milano-Bicocca, Milan, Italy

**Keywords:** academic self-efficacy beliefs, scale development and validation, measurement invariance, university students, academic experiences, students’ performance

## Abstract

Academic self-efficacy beliefs influence students’ academic and career choices, as well as motivational factors and learning strategies promoting effective academic success. Nevertheless, few studies have focused on the academic self-efficacy of university students in comparison to students at other levels. Furthermore, extant measures present several limitations. The first aim of this study was to develop a reliable and valid scale assessing university students’ self-efficacy beliefs in managing academic tasks. The second aim was to investigate differences in academic self-efficacy due to gender, years of enrollment, and student status. The study involved 831 students (age *M* = 21.09 years; *SD* = 1.34 years; 66.3% women) enrolled in undergraduate programs. Indicators of academic experiences and performance (i.e., number of exams passed and average exam rating) were collected. A new scale measuring students’ academic self-efficacy beliefs was administered. Results from a preliminary Exploratory Factor Analysis were consistently supported by findings from a Confirmatory Factor Analysis. Multigroup CFA supported the presence of measurement invariance. Analyses revealed that the new scale has eight factors: “Planning Academic Activities,” “Learning Strategies,” “Information Retrieval,” “Working in Groups,” “Management of Relationships with Teachers,” “Managing Lessons,” “Stress Management,” and “Thesis Work.” Self-efficacy dimensions showed significant relations with academic experiences and students’ performance indicators, as well as differences due to gender, years of enrollment, and student status. Findings are discussed in terms of practical implications for the implementation of intervention programs aimed at fostering self-efficacy beliefs and academic success.

## Introduction

Perceived self-efficacy refers to personal beliefs on the ability to maintain established goals and perform successful actions (Bandura, 1997), particularly in difficult moments (McGeown et al., 2014). Self-efficacy could concern a general or a specific belief: the first refers to a general perceived ability to face stressful conditions, while the second refers to a particular context or situation. This paper focuses on specific self-efficacy beliefs related to the academic field defined as students’ perceived abilities to successfully master different curricular areas, to self-regulate learning activities, and to manage relationships with teachers and peers (Bandura et al., 1996; Bassi et al., 2007). Academic self-efficacy has a significant and strong relationship with academic achievement (e.g., Pajares and Urdan, 2005; Ferla et al., 2009; Brausch, 2011), as cognitive and learning skills are necessary but not always sufficient (Bandura, 1997). Effective functioning requires two components, skills and efficacy beliefs to execute them appropriately, that act upon one another in a reciprocal fashion. Bandura referred this as a “reciprocal causation” in which the functioning of one component depends, in part, upon the functioning of the other (Bandura, 1997). In this way, students with high levels of self-efficacy can transform troubles into opportunities, think strategically to solve their difficulties and feel in control of a majority of stressors in their lives (Bandura, 1997). Academic self-efficacy is particularly salient when students have to cope with performance adversity or failure (Bong and Skaalvik, 2003). Research has also indicated that there is a positive relationship between academic self-efficacy beliefs and motivation (Ommundsen et al., 2005), in particular with intrinsic motivation (Walker et al., 2006). Self-efficacy beliefs contribute to motivation in several ways: determining the goals people set for themselves, how much effort they expend, how long they persevere in the face of difficulties, and their resilience to failures (Bandura, 1994). Moreover, Bandura (1997) declared that people derive information to evaluate efficacy beliefs from four primary sources: (1) mastery experiences; (2) vicarious experiences; (3) forms of persuasion, both verbal and otherwise; and (4) “physiological and affective states from which people partly judge their capableness, strength, and vulnerability to dysfunction.” Furthermore, self-efficacy beliefs foster positive social and supportive relationships (Bandura et al., 1996) that may help to reduce anxiety and to improve stress management (Mayer et al., 2002), especially in challenging contexts, such as the academic environment.

Studies on university students are less numerous compared to those on younger students. Nevertheless, they clearly show that those who feel more competent are more self-determined, demonstrating more effective self-regulation strategies and higher persistence to maintain their academic goals (Ryan and Deci, 2006). Conversely, students with low levels of academic self-efficacy have less motivation and are more passive and disengaged (Vallerand, 2000; Komarraju and Dial, 2014). Academic self-efficacy is also a significant predictor of university students’ course selection (Britner and Pajares, 2006; Komarraju and Dial, 2014), academic continuance, and achievement (Britner and Pajares, 2006). In particular, a study conducted by Amini (2002) showed that 21% of academic achievement was explained by students’ academic self-efficacy, whereas other studies showed a relationship with academic persistence (Robbins et al., 2004; Gore, 2006), and final GPA (Robbins et al., 2004).

In literature, it was also possible to find studies about self-efficacy in specific domains, such as in the health, sports, and educational fields. For instance, the Cardiovascular Management Self-efficacy by Steca et al. (2015) is an instrument to monitor differences during interventions to improve good disease management. About sports, Feltz and Lirgg (2001) made a literature review of individual beliefs, team beliefs, and coaches’ and leaders’ beliefs in sports, to better understand the dynamics of teams. Gould et al. (1999) found that personal and team self-efficacies were one of the most important elements to influence Olympic performances. In the educational field, Caprara et al. (2006) analyzed the Teachers’ self-efficacy beliefs as determinants of their job satisfaction and students’ academic achievement.

Specific self-efficacy beliefs are not stable, but, in line with Bandura’s thought, they are workable and flexible as they are strongly influenced by multiple sources (Bandura, 1997). A wide variety of educational, psychological, and pedagogical interventions are aimed at improving students’ self-efficacy beliefs for their beneficial effects on numerous outcomes (Lane et al., 2004). Indeed, it is critical to validly and reliably measure academic self-efficacy beliefs to set and evaluate interventions. A multifaced instrument that is specifically designed to measure numerous areas related to the activities of undergraduate students (e.g., individual effort and self-management skills, learning strategies, social, leisure and extracurricular activities, interaction with peers and teachers) would further facilitate substantive research in this area (Bandura et al., 1996; Cheung and Kwok, 1998; Amenkhienan and Kogan, 2004). Unfortunately, scales currently used to measure self-efficacy beliefs in university students present several limitations. For instance, Advance Care Planning Self-Efficacy focuses only on one aspect of academic self-efficacy beliefs, namely students’ concern about planning and their ability to do so. Furthermore, as underlined by the authors, it can be used only for a specific sample: medical doctors or those who need to initiate an Advance Care Planning (ACP) conversation (Baughman et al., 2015). Similar limitations characterize the Self-Efficacy Scale in Academic Behaviors in Students of Social Science by Blanco et al. (2013) and the Engineering Self-Efficacy Scales by Mamaril (2014). Scales referring to more than one aspect are often very short, like the Student Self-Report of Academic Self-Efficacy Scale by Hoover-Dempsey and Sandler (2005) composed of only three items, which are unable to cover the complexity of academic self-efficacy beliefs. As a consequence, more than one instrument has to be used to have a full measurement of self-efficacy beliefs in the university field. This could present difficulties because students consider answering several questionnaires as burdensome and not inherent to their academic path. Moreover, apart the Engineering Self-Efficacy Scales by Mamaril (2014), the other instruments presented lack of a psychometrics validation. Therefore, a significant limitation in this area is the missingness of a valid and reliable instrument that assesses the multidimensional nature of self-efficacy in the context of an academic setting. It is imperative that a self-report scale that reflects the various facets of self-efficacy be available. Even at the detriment of a lower specificity compared to other scales, only a multifaceted scale may guarantee the possibility to compare students from different degree programs adding information on differences in self-efficacy beliefs and on the differential effectiveness that interventions may have on students from different curricula and conditions.

### Current Study

Given the limitations of extant scales and considering the need for a multifaceted instrument, the main aim of this study was to develop a reliable and valid scale assessing university students’ self-efficacy beliefs in managing academic tasks; moreover, we also aimed to investigate differences in self-efficacy beliefs due to gender, years of enrollment in an undergraduate degree course, and supplementary-year student status. Finally, the final aim was to show how to use the scale to develop students’ profiles.

In relation to the first aim, we tested the dimensionality of the scale. Various researchers suggested developing a scale starting with Exploratory Factor Analysis (EFA) to assess factor structure and to refine the item pool; EFA should be followed by Confirmatory Factor Analysis (CFA) using a different sample to confirm the measure’s factor-structure and psychometric properties (Costello and Osborne, 2005; Henson and Roberts, 2006; Worthington and Whittaker, 2006; Cabrera-Nguyen, 2010). Worthington and Whittaker (2006) highlighted that EFA followed by CFA is the most common approach to scale development and validation. Our sample size was sufficient to utilize EFA in a random split-half of the sample (named “development sample”); the results were then verified using CFA in the second split-half (named “validation sample”). Further, we tested measurement invariance on the whole sample by a mean of Multi-group CFA. We then explored the internal consistency of the scale and examined the associations of self-efficacy factors with indicators of academic experiences and students’ performance, namely the number of exams passed and average exam rating. We hypothesized that self-efficacy factors were significantly and negatively associated with negative experiences during academic life as students feeling more able to manage academic tasks should have better management of their academic lives. Furthermore, we hypothesized significant and positive relations between self-efficacy beliefs and indicators of students’ performance. These relations were tested considering students at their different years of the undergraduate program separately to examine these associations more carefully. In line with Bandura’s previous studies (1997), we expected that self-efficacy beliefs would be more predictive of the students’ career achievements. In that sense, one of our aims was to investigate which particular domain of self-efficacy belief is the best support for the personal competences useful in academia. In line with the Life Design approach (Savickas et al., 2009) and with the study of Azizli et al. (2015), we hypothesized that the ability to plan activities could be one of the most helpful self-efficacy beliefs to achieve career goals.

In relation to the second aim, related to the investigation of differences in self-efficacy beliefs due to gender, years of enrollment in an undergraduate degree course, and supplementary-year student status, we firstly conducted the relative tests for measurement invariance. In line with Ceci et al. (2014) and Voyer and Voyer (2014), we hypothesized that women would show better competences helpful for academia. We also considered it interesting to analyze the presence or absence of gender differences in other competences, in particular with stress management, given women generally show higher levels of stress (Cohen and Janicki-Deverts, 2012). Lastly, we were interested in exploring to what degree self-efficacy competence is present in students in line with their exam schedule vs. supplementary-year students, given that no study exists yet on this issue. We expected that students in line with the exam schedule had a higher general level of self-efficacy beliefs than the supplementary-year students. In addition, we hypothesized that the university and the academic context played an important role in improving the level of self-efficacy, establishing higher and higher demands as the degree course progresses; as suggested by Bandura (1997), in fact, mastery experience or, in other words, performing a task successfully, is the most effective way to strength self-efficacy beliefs. We hypothesized a higher level of self-efficacy competence in the second or third enrollment year.

The final aim of this study was to show how to use the scale to develop students’ profiles consisting of perceived “strengths” and “weaknesses” that match their self-efficacy beliefs, which correspond to the areas in which students deem themselves more or less able to behave effectively. The profiles may be used in intervention programs aimed at fostering self-efficacy beliefs and academic success, starting from a precise and reliable assessment.

## Materials and Methods

### Participants and Study Design

The participants were undergraduate students recruited from 24 Italian universities. The inclusion criteria were as follows: (a) no other previous degree, (b) age under 26 years, and (c) fluent in the Italian language. Eligible students received written information about the study and signed an informed consent form; participation was voluntary and provided no remuneration. The students filled the instruments during the weeks of teaching, in a 20-min session during a lesson. The study has a cross-sectional design and was approved by the Ethics Committees of the university that conducted the research.

We recruited 831 students from 13 faculties or departments and 73 courses. Participants were mostly women (*n* = 551, 66.3%), with a mean age of 21.09 years (*SD* = 1.34; range: 19–25 years). The majority (*n* = 369, 44.4%) were psychology students, followed by economics (*n* = 135, 16.2%) and engineering ones (*n* = 79, 9.5%); the rest (*n* = 248, 29.9%) were from other 10 faculties or departments. Two hundred and thirty-six students (28.4%) were enrolled in the first year of the degree courses, 108 in the second (13.0%) and 407 in the third (49.1%), while 78 (9.4%) were supplementary-year students (2 missing data). Finally, 380 students (45.7%) declared that they were preparing their theses.

### Variables and Instruments

#### The Academic Self-Efficacy Scale

By following Bandura’s guidelines exactly (Bandura, 2006), we conducted a preliminary study in one of the faculties participating in the research; we involved a teacher and nine volunteer students enrolled in a 3-year undergraduate degree program with three students for each year of the degree course. In this phase, participants had to answer open questions related to tasks and problems that students could encounter in managing academic demands [i.e., What are the tasks or activities that a student, as you or someone like you, have to do to fulfill to successfully manage academic demands? What are the problems that could encounter in managing academic demands? What are the ways (tasks or activities to do) out of such problems?]; the teacher had to answer these questions from their point of view considering tasks and problems that students could encounter in managing academic demands. In the same phase, they were asked to imagine how they could face these tasks and problems. This procedure allowed us to identify activities and situations that students frequently have to manage in their academic lives, as well as successful behaviors.

The behaviors that emerged were then transformed into items to measure students’ self-efficacy beliefs. These items theoretically measure: “planning,” namely students’ beliefs that they can carefully plan and organize tasks, activities, and goals to achieve about academic demands; “information retrieval,” reflecting students’ perceptions of their ability to regularly collect information about the course of study; “learning strategies,” namely students’ perceptions of their abilities to strictly comply with study responsibilities and rework the concepts of the field of study; “relationships,” namely students’ beliefs that they are able to work in groups using appropriate study strategies; “stress management,” reflecting students’ perceptions of their abilities to adequately control negative emotions about exam-taking; “thesis work,” namely students’ perceptions regarding their abilities to strictly comply with thesis writing. After this step, a teacher and three volunteer students in the same department verified the comprehensibility of the items. The teacher and students involved in this phase were different from those involved in the previous one. They reported some suggestions for the items to be more easily understood by other students. Some of these changes required inserting specific examples referring to information retrieval (e.g., “opening times, ways of contacting offices”) or to learning strategy (e.g., “relating concepts together, making outlines, exam review”). At the end of these phases, 44 items were developed: 37 general items for all students enrolled in an undergraduate program, and 7 for students involved in the final thesis preparation. For each item, participants rated the strength of their beliefs on a 5-point response format ranging from 1 (perceived inability) to 5 (complete self-assurance in one’s ability).

None of the students involved in the construction of the scale took part in the subsequent phase of the study.

#### Academic Experiences

A pool of 24 questions developed by the authors was used to measure four kinds of experiences relating to academic experiences: planning experiences (11 items, referring to how many times the respondent could have problems passing exams because of several reasons, α = 0.78, example item “How many times have you failed an exam because you did not sort what you had to study in the time you had left?); finding information experiences (seven items, referring to how many times the respondent encountered problems because of several reasons such as for example not paying attention to warnings displayed on the bulletin board, α = 0.68, example item “How many times have you had problems because you did not find out about the exam format ahead of time?”); learning experiences (three items, referring to how many times the respondent could have problems getting to the exam unprepared because of several reasons such as not using appropriate learning strategies or focusing on less relevant concepts of a field of study, α = 0.64, example item “How many times have you focused on less relevant concepts in what you were studying and overlooked more important ones?); stress (three items, referring to how many times the respondent could have difficulty taking an exam because of several reasons such as being overwhelmed by anxiety, α = 0.79, example item “How many times have you skipped an exam because you were overcome with anxiety?”). All the items were rated using a 5-point Likert scale, ranging from “never” (1) to “very often” (5); the scores were calculated as mean item scores, where higher scores indicate more negative academic experiences.

#### Students’ Performance Indicators

Indicators of students’ performance were collected for each participant and are relative to the number of exams passed, the number of exams required each year by rules of the degree course, and the average exam rating. Given there are different rules for different degree courses (i.e., the number of exams for each degree course, the number of exams due each year for each degree course), the number of exams passed and the number of exams required each year by rules of the degree course were used to calculate a proportion of exams passed per participant; this new variable was used in the subsequent analyses. Students were also asked to indicate information about the year of the degree course in which they were enrolled and their status (in line with the exam schedule vs. supplementary-year students).

### Data Analysis

The items of the new scale were preliminarily submitted to analyses to check the normal distribution by calculating mean, standard deviation, and indices of skewness and kurtosis; West et al. (1995) recommend concern if skewness > |2| and kurtosis > | 7|.

#### Students Not Involved in the Thesis Work

For students not involved in their thesis work, the total sample was later randomly divided into two halves. The first sample was used to perform an EFA (DEVELOPMENT SAMPLE, *n* = 414) and the second was used to perform a CFA for validating the EFA symptom structure (VALIDATION SAMPLE, *n* = 417). To avoid problems with missing data, the 7 items developed for students involved in thesis work were excluded from these analyses, because these items were filled out only by students in the situation proposed.

On DEVELOPMENT SAMPLE, the Kaiser Meyer Olkin (KMO) and Bartlett’s test of sphericity were run to be sure that the correlation matrix could be subjected to analyses (KMO should be >0.5; Bartlett’s test of sphericity should be significant). Horn’s method of parallel analysis was used to identify the number of factors to be extracted using EFA (Horn, 1965). Horn’s method was chosen because of its merits as an objective test for identifying the dimensionality of multivariate data (Hubbard and Allen, 1987). Horn’s method is, in fact, more accurate than the Cattell scree test or the Kaiser-Guttman criteria: judging the elbow of a scree plot could reflect a sampling error, while an eigenvalue greater than one tends to retain too many factors (Hubbard and Allen, 1987; Netemeyer et al., 2003). EFA with the Promax oblique rotation was used to analyze the items on the Academic Self-Efficacy Scale. Oblique rotation was used because the factors extracted from the Academic Self-efficacy Scale are likely to correlate with each other. In the first step, all 37 general items were included. Subsequent factor analyses were conducted in a stepwise fashion to eliminate items until a stable factor solution emerged. Items that had a factor loading < 0.32 were excluded, and, after the first step, items that loaded at >0.32 on more than one factor were excluded. Loadings in the 0.32 range or above are generally considered the cut-off on substantial loadings (Comrey and Lee, 1992).

On VALIDATION SAMPLE, CFA was conducted and Maximum Likelihood (ML) was used as an estimation method. Hu and Bentler’s guidelines for various fit (1999) indices were used to determine whether the expected model fits the data. The chi-square test statistic was used but considering the sensitivity of the chi-square statistic to the sample size other goodness of fit indices were considered as the root-mean square error of approximation (RMSEA) and the standardized root-mean-square residual (SRMR). RMSEA and SRMR ≤ 0.08 were interpreted as a reasonable fit. Moreover, it would be desirable to additionally report the comparative fit index (CFI) and the Tucker Lewis index (TLI). However, cases where the RMSEA of the null model is <0.158 render the CFI and the TLI non-interpretable (Kenny, 2020). Hence, such incremental indices were considered only when the null model RMSEA was above.158. CFI and TLI ≥ 0.90 were interpreted as reasonable.

#### Students Involved in the Thesis Work

With the same procedure indicated above, a separate EFA in a random subsample followed by a CFA in the other subsample were performed to test the dimensionality of the seven items developed for students involved in thesis work. Moreover, on the total subsample of students involved in thesis preparation, an overall CFA was performed to test the model resulted from the analyses on the whole set of 37 items, adding the seven items developed for students involved in thesis work. For both students involved and those not involved in thesis work, Cronbach’s alpha, McDonald’s (1999) omega, and the items’ inter-correlations coefficients were performed on the total sample to examine internal consistency. Cronbach’s Alpha and McDonald’s omega below 0.60 are unacceptable (Nunnally and Bernstein, 1994), whereas the items’ inter-correlations coefficients that are higher than 0.30 are adequate (Nunnally and Bernstein, 1994).

#### Validity, Measurement Invariance and Group Comparisons

To investigate the validity of the self-efficacy scale, we conducted correlations using all the scale scores computed as average item scores. For convergent validity, the relations among self-efficacy beliefs and academic experiences was assessed via Pearson correlation. Further, the relations among self-efficacy beliefs and students’ performance indicators were also tested. Students at different years of the undergraduate program were tested separately to consider these associations more carefully. Following guidelines by Cohen (1988), we interpreted correlations as measures of effect size. Correlations were considered weak (| 0.10| < *r* < |0.29|), moderate (|0.30| < *r* < |0.49|), or strong (|0.50| < *r* < |1|).

Furthermore, multi-group CFA were conducted on the whole sample to assess measurement-invariance (Blunch, 2012) for each of the three variables of interest: gender, status, and year of enrollment. Multi-group CFA were also conducted on the sample of 380 students preparing their thesis. Three different models were obtained and compared: (i) configural invariance, which served as a baseline model and where the structure is assumed to be the same in the various groups being compared (e.g., males vs. females); (ii) metric (or weak) invariance, where loadings are fixed to being equal across groups, and; (iii) scalar (or strong) invariance, where loadings and intercepts are fixed to be equal across groups. We considered metric and/or scalar invariance to be present when the corresponding models (ii and/or iii) fit the data, as well as model i (configural invariance), did. To compare the three models, we focused on the changes in RMSEA and SRMR (see also Lu et al., 2018; Zhao et al., 2019; Ma, 2020), since the χ2 difference test is too sensitive for the assessment of invariance with large samples (*N* > 300, Chen, 2007). Following Cheung and Rensvold (2002) and Chen (2007), we considered measurement invariance to be present when ΔRMSEA < 0.015 and ΔSRMR < 0.030. ΔCFI and ΔTLI were only reported if the null model RMSEA was < 0.158. Bayesian Information Criterion (BIC) values were also compared, with lower values indicating better fit and evidence of invariance (Cheung and Rensvold, 2002; Zhou et al., 2019). If both ii and iii forms of invariance were attained, we concluded that meaningful comparisons in the scores of the Academic Self-Efficacy Scale could be made for gender, and/or status, and/or year of enrollment. For such cases where invariance was assured, *t*-tests and univariate ANOVA were used to test the difference among profiles of the Academic Self-Efficacy Scale due to gender, year of enrollment in an undergraduate degree course, and students in line with the exam schedule vs. supplementary-year students (status).

Data analyses related to the normal distribution, EFA, Cronbach’s alpha, items’ inter-correlations, correlations, *t*-tests, and univariate ANOVA were performed using IBM SPSS Statistics (Version 22). Parallel analysis, CFA, and McDonald’s omega were performed using MPlus software (Version 7) (Muthén and Muthén, 1998-2010). Multi-group CFA were performed with R (Version 4.0.3) (R Core Team, 2020) and R studio (Version 1.3.1093) (RStudio Team, 2020) using the R package lavaan (Rosseel, 2012). Missing values were treated via listwise deletion in SPSS and full information ML estimation in Mplus and R.

## Results

### Preliminary Analysis

The average scores of the responses to the 44 items from all participants ranged from 2.32 to 4.32 (*SD*_MIN_ = 0.77-*SD*_MAX_ = 1.13). Furthermore, in line with recommendations by West et al. (1995), all the items showed an acceptable distribution; skewness and kurtosis showed no non-normally distributed items (Skewness_MIN_ = –1.25-Skewness_MAX_ = 0.64; Kurtosis_MIN_ = –0.73-Kurtosis_MAX_ = 1.12).

### Factor Structure of the Academic Self-Efficacy Scale. Exploratory Factor Analysis

Data from Development Sample and 37 general items were used in these analyses. The Bartlett’s sphericity test (χ*^2^* = 3628.64, *p* < 0.001) and the KMO = 0.85 have ensured that the correlation matrix could be subjected to factor analysis. The parallel analysis indicated that a seven-factor solution was the most appropriate. EFA was then conducted, with seven factors extracted. The initial pool of 37 general items, after subsequent factor analyses conducted in a stepwise fashion, was reduced to 30 (items are present in the Supplementary Materials). Four items were excluded because their loadings were lower than 0.32: “How well can you make friends with students who are stimulating for your degree course?”; “How good are you about consulting your representatives to find out about your rights?”; “How good are you at getting useful study advice by asking students who have already taken the tests?”; “How well can you gather useful study information by being present at other students’ exams?”. Three items were excluded because their loadings were above 0.32 on more than one factor: “How well can you critically judge the information other students give you?”; “How well can you get the materials you need on time to study for tests?”; “How well can you take advantage of appropriate and effective learning strategies (e.g., “relating concepts together, making outlines, exam review, etc.)?”.

The pattern of factor loadings from the seven-factor exploratory measurement model for the self-efficacy scale’s 30 items is given in Table 1.

**TABLE 1:** Item percentage of response frequency and factors loadings from the Exploratory Factor Analysis in DEVELOPMENT SAMPLE and Confirmatory Factor Analysis in VALIDATION SAMPLE.

How well can you…	*Development Sample*								*Validation Sample*	
	*% response*	*PAA*	*LS*	*IR*	*WG*	*MRT*	*SL*	*SM*	*% response*	*Loadings* ^a^
. keep with the study schedule you set up	99.52	**0.81**	–0.08	–0.08	0.05	0.00	0.00	–0.02	100	0.77***
. sort what you have to study in the time you have left to prepare for an exam	100	**0.79**	0.04	–0.01	–0.02	–0.01	–0.18	–0.02	100	0.71***
. keep up continuous study habits throughout the school year	99.76	**0.70**	–0.15	–0.02	–0.04	0.13	0.14	–0.13	99.76	0.73***
. organize your time in order to finish a paper by the deadline	99.03	**0.70**	0.05	0.07	–0.05	–0.09	–0.01	0.08	99.04	0.71***
. plan the number of exams you will take in each session based on how difficult they are	100	**0.53**	0.06	0.10	–0.03	–0.07	0.05	0.15	99.52	0.59***
. set achievable goals by knowing your abilities and your limitations	99.52	**0.39**	0.22	0.08	0.10	–0.04	0.01	0.11	99.76	0.54***
. make connections, analogies and distinctions among the various subjects you are taking	100	–0.08	**0.63**	0.08	–0.04	0.01	0.00	0.03	100	0.57***
. at the exam, convey in writing what you’d studied	99.28	0.06	**0.62**	–0.08	–0.04	–0.01	0.08	−−0.09	99.52	0.65***
. enhance your exam preparation with personalized, in-depth study	99.52	–0.04	**0.56**	0.05	0.05	0.08	–0.01	–0.11	99.52	0.47***
. adjust your way of expressing yourself according to the situation and the person you’re talking to	99.76	–0.07	**0.56**	–0.04	–0.02	–0.07	0.12	0.04	99.52	0.55***
. demonstrate your knowledge of that you’ve studied in an oral exam	98.79	0.11	**0.51**	0.00	0.11	–0.09	0.03	0.00	98.80	0.63***
. focus on the main points of what you are studying	100	0.01	**0.51**	–0.01	0.01	0.05	–0.04	0.13	99.52	0.55***
. get the information you need about administrative offices (opening times, how to contact them.)	100	–0.09	–0.12	**0.76**	–0.05	–0.02	0.11	0.03	100	0.67***
. get information from the university website	99.76	–0.03	0.10	**0.71**	–0.06	–0.01	–0.13	0.06	99.76	0.69***
. regularly check the departmental notice board to get information about your degree course	100	0.10	0.00	**0.64**	0.00	0.11	–0.07	–0.10	100	0.64***
. get information on exam formats ahead of time	99.52	0.01	0.15	**0.46**	–0.07	0.00	0.10	–0.06	99.52	0.61***
. sign up for exams within the established timeline	99.76	0.04	–0.23	**0.42**	0.08	–0.13	0.31	0.11	99.76	0.47***
. find out ahead of time if there are any prerequisite exams to take in your degree course before beginning other courses	99.52	0.07	0.08	**0.42**	0.03	0.05	–0.04	–0.11	98.80	0.47***
. start efficient study groups	99.03	0.02	–0.07	–0.07	**0.82**	–0.01	0.08	0.00	100	0.76***
. use good group study strategies (quiz each other, etc.)	99.52	0.00	0.09	–0.01	**0.80**	–0.07	–0.08	–0.06	99.76	0.69***
. work together productively by defining specific goals and tasks	99.03	–0.04	0.00	–0.01	**0.67**	0.13	0.03	0.06	99.76	0.82***
. raise your hand to ask the professor to explain parts of the lesson that you don’t understand	99.76	–0.03	–0.01	0.06	–0.05	**0.84**	0.00	0.02	99.76	0.82***
. participate actively in in-class discussion	99.76	0.00	0.00	–0.08	0.05	**0.71**	0.00	0.10	99.76	0.77***
. go to your professors to get useful information on courses	98.79	0.06	0.03	0.16	0.16	**0.36**	0.05	–0.04	99.52	0.43***
. stay focused in class even when is is noisy or crowded	99.76	0.10	0.09	–0.09	–0.18	0.11	**0.55**	–0.01	99.52	0.61***
. attend class regularly even when the exam session approaches	100	–0.18	0.08	0.15	0.11	–0.06	**0.51**	0.02	99.76	0.43***
. take clear, useful notes in class	99.76	0.13	0.06	0.01	0.09	–0.03	**0.49**	–0.11	100	0.70***
. glean and reprocess the essential points in a lecture	99.76	–0.02	0.30	–0.06	–0.01	0.08	**0.44**	0.06	99.52	0.74***
. keep exam anxiety under control	100	0.01	0.02	–0.07	–0.08	0.01	0.07	**0.69**	100	0.87***
. avoid getting discouraged when you fail an exam	98.79	0.03	–0.02	0.03	0.07	0.10	–0.12	**0.67**	99.04	0.61***

***p < 0.001. PAA, “Planning Academic Activities”; LS, “Learning Strategies”; IR, “Information Retrieval”; WG, “Working in Groups”; MRT, “Management of Relationships with Teachers”; SL, “Skills for Lessons”; SM, “Stress Management.” ^a^Items selected to load on CFA factors are based on EFA loadings. Bold items indicate factor membership.

The first extracted factor explains 9.32% of the variance. It showed loadings from six items assessing students’ beliefs regarding their ability to carefully organize time, plan the number of exams, sort the study material, maintain a steady pace of study, and establish achievable goals concerning academic demands. This factor can be called “Planning Academic Activities.” The second extracted factor explains 7.56% of the variance. It showed strong loadings from six items assessing students’ beliefs regarding their ability to strictly comply with study tasks such as focus primarily on core concepts, create connections, enhance exam preparation, adequately reprocess and explain the study material. This factor can be called “Learning Strategies.” The third extracted factor explains 7.29% of the variance. It showed loadings from six items assessing students’ beliefs regarding their ability to regularly collect information about the course of study and the various examinations through the different sources available such as notice boards, administrative offices, and websites. This factor can be called “Information Retrieval.” The fourth extracted factor explains 6.25% of the variance. It showed strong loadings from three items assessing students’ beliefs regarding their ability to be good to create study groups and use adequate and productive strategies in this context. This factor can be called “Working in Groups.” The fifth extracted factor explains 4.78% of the variance. It showed loadings from three items assessing students’ beliefs regarding their ability to take an active role in classroom discussion, and refer to teachers for more information and clarification about courses and lessons. This factor can be called “Management of Relationships with Teachers.” The sixth extracted factor explains 4.28% of the variance. It showed strong loadings from four items assessing students’ beliefs regarding their ability to attend classes, keep focused even in challenging circumstances, take clear and helpful notes, and reprocess the main parts of a lesson. This factor can be called “Skills for lessons.” The seventh and final extracted factor explains 3.51% of the variance. It consisted chiefly of two items assessing students’ beliefs regarding their ability to adequately control exam-related anxiety, and discouragement after a failed exam. An appropriate name for this factor might be “Stress Management.” The total variance explained by the seven factors extracted was 43.00%.

As shown in Table 1, no item displays a loading lower than 0.32. The extent of cross-loading between factors was moderate; the size of this secondary loading was usually small, below 0.32.

### Factor Structure of the Academic Self-Efficacy Scale. Confirmatory Factor Analysis

Confirmatory factor analysis was conducted separately on data from Validation Sample using the 30 items; item selection to load on CFA factors was based on EFA loadings. Table 1 presents the standardized factor loadings in Validation Sample. The fit of the CFA model to the data from the 417 students was acceptable [χ*^2^*(384) = 930.206, *p* < 0.001; RMSEA = 0.058; SRMR = 0.067]; we therefore examined the RMSEA of the null model and found RMSEA null = 0.145. Therefore, we refrained from reporting the CFI or other incremental fit indices. Loadings from the CFA were comparable with those found in the EFA, identifying the seven factors.

### Academic Self-Efficacy Scale Related to Thesis Work

Data from the 380 students that filled out the seven items developed for those involved in thesis preparation were used in these analyses. The sample was randomly split into two subsamples.

The first subsample (*n* = 190) was used to perform an EFA to test the dimensionality of the scale. The Bartlett’s sphericity test (χ*^2^* = 678.46, *p* < 0.001) and the KMO = 0.87 have ensured that the correlation matrix could be subjected to factor analysis.

The pattern of factor loadings from the one-factor exploratory measurement model for the self-efficacy scale’s 7 items is given in Table 2. The extracted factor explains 53.51% of the variance. It showed loadings from seven items assessing students’ beliefs regarding their ability to strictly meet all graduation deadlines, to design, find, organize and regularly work to complete a good project for the thesis. This factor might be called “Thesis Work.” As shown in Table 2, all items display adequate loadings, higher than 0.32.

**TABLE 2:** Item percentage of response frequency and factors loadings from the Exploratory Factor Analysis in a random subsample and Confirmatory Factor Analysis in the other subsample for the seven items related to the preparation of the thesis.

How well can you…	*EFA SUBSAMPLE*		*CFA SUBSAMPLE*	
	*% response*	*Loadings*	*% response*	*Loadings*
. select what is useful from all your research to write your thesis	99.47	0.80	98.95	0.74***
. use a clear and coherent structure to organize your research material for the thesis	98.95	0.76	98.42	0.69***
. devise a good project for your thesis	99.47	0.66	100	0.65***
. make good use of your advisor’s suggestions to write your thesis	100	0.65	98.95	0.51***
. work continually in order to finish your thesis in time	100	0.65	98.95	0.65***
. use library resources to find materials for your thesis	100	0.62	98.42	0.68***
. respect all graduation deadlines (getting a thesis advisor, graduation application, handing in documents.)	100	0.61	98.95	0.60***

***p < 0.001.

Confirmatory factor analysis was conducted separately on the other subsample. Table 2 presents the standardized factor loadings in these subsamples. The fit of the CFA model to the data from the 190 students was acceptable [χ*^2^*(14) = 30.137, *p* < 0.01; CFI = 0.96, TLI = 0.94; RMSEA = 0.078; SRMR = 0.038]. Loadings from the CFA were comparable with those found in the EFA, identifying one factor.

### Factor Structure of the Academic Self-Efficacy Scale and the Academic Self-Efficacy Scale Related to Thesis Work

On the data from the 380 students that filled out the seven items developed for those involved in thesis preparation, an overall CFA was performed to test a model with eight factors, seven from the analyses on the whole set of 30 items, adding the “Thesis Work” factor. The fit of the CFA model to the data was acceptable [χ*^2^*(601) = 1280.146, *p* < 0.001; RMSEA = 0.055; SRMR = 0.066]. We therefore examined the RMSEA of the null model and found RMSEA null = 0.133. Therefore, we refrained from reporting the CFI or other incremental fit indices. Loadings from the CFA were comparable with those found in the previous CFA.

### Reliability of the Academic Self-Efficacy Scale and Correlations Among Subscales

For each subscale, the score was calculated by computing the average score across items within a subscale (ranging from 1 to 5). All the factor scores showed an acceptable distribution; skewness and kurtosis showed normal distribution (Skewness_MIN_ = –0.16-Skewness_MAX_ = 0.54; Kurtosis_MIN_ = –0.39-Kurtosis_MAX_ = 0.75).

The analysis of reliability performed on the data collected from all participants (831 students for the Academic Self-efficacy Scale and 380 students for the Academic Self-efficacy Scale Related to Thesis Work) showed that the scale has adequate internal consistency for all factors. All Cronbach’s alpha and McDonald’s omega were adequate: “Planning Academic Activities” = α = 0.83, ω = 0.83; “Learning Strategies” = α = 0.75, ω = 0.75; “Information Retrieval” = α = 0.76, ω = 0.76; “Working in Groups” = α = 0.80, ω = 0.80; “Management of Relationships with Teachers” = α = 0.71, ω = 0.73; “Skills for lessons” = α = 0.68, ω = 0.70; “Stress Management” = α = 0.65, ω = 0.65; “Thesis Work” = α = 0.84, ω = 0.85. Moreover, the inter-correlations coefficients of items were all larger than.37, indicating adequate internal consistency.

As shown in Table 3, the self-efficacy factors were all positively and significantly correlated apart from “Information Retrieval” and “Stress Management,” which were shown to be uncorrelated.

**TABLE 3:** Pearson correlations among Academic Self-efficacy factors and indicators of students’ academic experiences and performance.

	PAA	LS	IR	WG	MRT	SL	SM	MTW
LS	0.41***	1						
IR	0.34***	0.23***	1					
WG	0.22***	0.23***	0.10**	1				
MRT	0.28***	0.40***	0.22***	0.32***	1			
SL	0.44***	0.47***	0.33***	0.12**	0.33***	1		
SM	0.13***	0.27***	0.00	0.13***	0.20***	0.11**	1	
MTW	0.47***	0.45***	0.35***	0.25***	0.41***	0.38***	0.17**	1
Planning experiences	−0.53**	−0.31**	−0.19*	–0.12	−0.20*	−0.38**	–0.05	–0.16
Finding information experiences	−0.32**	−0.25**	−0.41**	0.17	–0.15	−0.38**	0.17*	−0.38*
Learning experiences	−0.50**	−0.34**	–0.14	0.07	–0.08	−0.37**	–0.02	–0.18
Stress experiences	−0.42**	–0.17	−0.20*	−0.24**	–0.12	–0.08	−0.37**	0.04
First year Proportion of exams passed	0.27**	0.05	0.12	0.09	0.14*	0.13*	0.01	−
Average exam rating	0.30**	0.22**	0.05	0.09	0.07	0.14*	0.00	−
Second year Proportion of exams passed	0.23*	0.34**	0.07	0.08	0.02	0.13	0.02	−
Average exam rating	0.21*	0.32**	–0.03	0.14	0.23*	0.23*	–0.09	−
Third year Proportion of exams passed	0.35**	0.19**	0.10	0.11**	0.23**	0.13*	0.06	0.28***
Average exam rating	0.40**	0.35**	0.13**	–0.09	0.23**	0.27**	–0.03	0.21***

*p < 0.05; **p < 0.01; ***p < 0.001. PAA, “Planning Academic Activities”; LS, “Learning Strategies”; IR, “Information Retrieval”; WG, “Working in Groups”; MRT, “Management of Relationships with Teachers”; SL, “Skills for Lessons”; SM, “Stress Management”; MTW, “Management of Thesis Work.”

### Measurement Invariance of the Academic Self-Efficacy Scale

Multigroup Confirmatory Factor analyses to test for measurement invariance showed that for both the whole sample (seven factors) and the sample of 380 students preparing their thesis (eight factors), measurement invariance could be deemed as present. Indeed, as Table 4 shows, changes in RMSEA never exceeded 0.011, SRMR never exceeded 0.007, and that the BIC of the most parsimonious model (e.g., scalar invariance vs. metric invariance) were always the lowest. Hence, all comparisons for gender, status, and year of enrollment can be made (see below).

**TABLE 4:** Fit indices for the assessment of measurement invariance.

	chisq	df	rmsea	srmr	bic
Configural (gender – 7 factors)	1600.091	768	0.051095	0.061165	61964.070
Metric (gender – 7 factors)	1633.939	791	0.050674	0.062409	61843.330
Scalar (gender – 7 factors)	1762.909	814	0.053	0.06438	61817.710
Configural (status – 7 factors)	1752.759	768	0.055619	0.061611	62318.070
Metric (status – 7 factors)	1797.327	791	0.055401	0.06253	62208.070
Scalar (status – 7 factors)	1837.998	814	0.05509	0.062632	62094.180
Configural (year – 7 factors)	2077.993	1152	0.058156	0.069731	53771.700
Metric (year – 7 factors)	2175.298	1198	0.058587	0.073902	53566.810
Scalar (year – 7 factors)	2331.973	1244	0.060662	0.07578	53421.290
Configural (gender – 8 factors)	2030.765	1202	0.06024	0.075723	35528.910
Metric (gender – 8 factors)	2067.303	1231	0.059797	0.077872	35393.180
Scalar (gender – 8 factors)	2153.694	1260	0.061099	0.079856	35307.310
Configural (status – 8 factors)	2389.350	1202	0.072295	0.076482	35504.680
Metric (status – 8 factors)	2451.060	1231	0.072415	0.078845	35394.280
Scalar (status – 8 factors)	2486.359	1260	0.071762	0.07892	35257.470

### Correlations of Academic Self-Efficacy Factors With Indicators of Academic Experiences and Performance

We examined the correlations of the Academic Self-efficacy subscales with academic experiences and performance. As shown in Table 3, “Planning Academic Activities” was strongly and negatively correlated to negative experiences in planning and learning, and moderately and negatively associated with negative experiences in finding information and stress. “Learning Strategies” was moderately and negatively correlated to negative experiences in planning and learning, and weakly and negatively associated with negative experiences in finding information. “Information Retrieval” was moderately and negatively correlated to negative experiences in finding information, and weakly and negatively associated with negative experiences in planning and stress. “Working in Groups” was weakly and negatively correlated to negative experiences in stress. “Management of Relationships with Teachers” was weakly and negatively correlated to negative experiences in planning. “Skills for lessons” was moderately and negatively correlated to negative experiences in planning, finding information, and learning. “Stress Management” was moderately and negatively correlated to negative experiences in stress, while it was weakly and positively associated with negative experiences in finding information. “Management of Thesis Work” was moderately and negatively correlated to negative experiences in finding information.

Moreover, we examined the correlations of the Academic Self-efficacy subscales with indicators of students’ performance. The correlations were tested considering students at different years of the undergraduate program separately. As shown in Table 3, for the group of first year undergraduates, the proportion of exams passed was positively and weakly correlated to the “Planning Academic Activities,” “Management of Relationships with Teachers,” and “Skills for Lessons” subscales. The average exam rating was positively and moderately correlated to “Planning Academic Activities,” and weakly to the “Learning Strategies,” and “Skills for Lessons” subscales. For the group of second year undergraduates, the proportion of exams passed was positively and moderately correlated to “Learning Strategies,” and weakly to “Planning Academic Activities.” The average exam rating was positively and moderately correlated to “Learning Strategies,” and weakly to the “Planning Academic Activities,” “Management of Relationships with Teachers,” and “Skills for Lessons” subscales. For the group of third year undergraduates, the proportion of exams passed was positively and moderately correlated to “Planning Academic Activities,” and weakly to the “Learning Strategies,” “Working in Groups,” “Management of Relationships with Teachers,” “Skills for Lessons,” and “Management of Thesis Work” subscales. The average exam rating was positively and moderately correlated to “Planning Academic Activities” and “Learning Strategies,” and weakly associated with the “Information Retrieval,” “Management of Relationships with Teachers,” “Skills for Lessons,” and “Management of Thesis Work” subscales.

### Academic Self-Efficacy Scale in Measuring Strengths vs. Weaknesses

**FIGURE 1:**
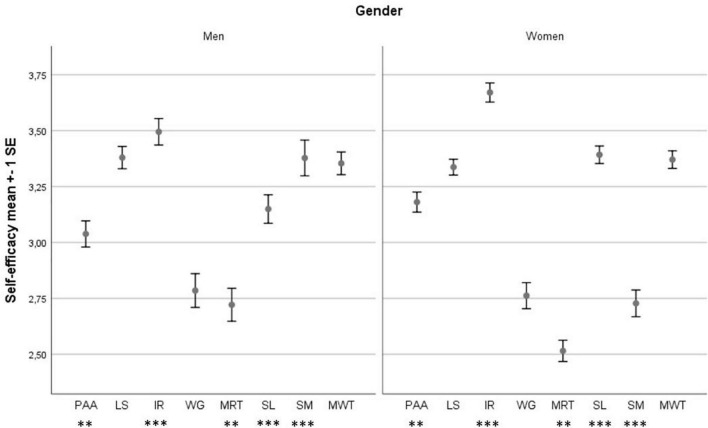
Mean levels of the Academic Self-efficacy factors for men and women and *t*-test results. **p < 0.01; ***p < 0.001; PAA, “Planning Academic Activities”; LS, “Learning Strategies”; IR, “Information Retrieval”; WG, “Working in Groups”; MRT, “Management of Relationships with Teachers”; SL, “Skills for Lessons”; SM, “Stress Management”; MWT, “Management of Thesis Work.”

Assessing self-efficacy beliefs allows us to develop profiles consisting of subjectively defined “strengths” and “weaknesses,” which reflect the areas in which students consider themselves more or less able to act effectively. Figure 1 shows mean values of Academic Self-efficacy for the 831 students for the Academic Self-efficacy Scale and 380 students for the Academic Self-efficacy Scale Related to Thesis Work divided by gender. Both genders showed strengths in “Information Retrieval,” but weaknesses in “Management of Relationships with Teachers” and in “Working in Groups.” Furthermore, the results of the *t*-test showed a meaningful difference between male and female students in their levels of “Planning Academic Activities” [*t*(df = 828) = –2.64, *p* < 0.01, Cohen’s *d* = 0.19],“Information Retrieval” [*t*(df = 828) = –4.31, *p* < 0.001, Cohen’s *d* = 0.31],“Management of Relationships with Teachers” [*t*(df = 826) = 3.29, *p* < 0.01, Cohen’s *d* = 0.24], “Skills for Lessons” [*t*(df = 828) = –5.07, *p* < 0.001, Cohen’s *d* = 0.36], and “Stress Management” [*t*(df = 828) = 10.49, *p* < 0.001, Cohen’s *d* = 0.76].

**FIGURE 2:**
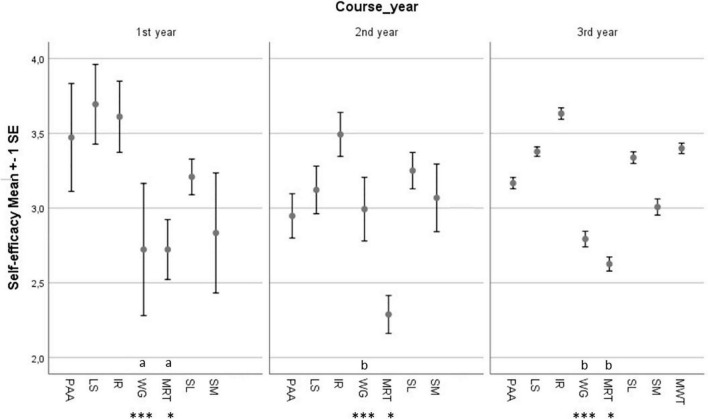
Mean levels of the Academic Self-efficacy factors for students at the first, second, and third year of the undergraduate degree course and results from univariate analysis of variance and *post hoc* comparisons based upon Tukey tests. *p < 0.05; ***p < 0.001; Different letters indicate significant differences among groups; PAA, “Planning Academic Activities”; LS, “Learning Strategies”; IR, “Information Retrieval”; WG, “Working in Groups”; MRT, “Management of Relationships with Teachers”; SL, “Skills for Lessons”; SM, “Stress Management.”

Figure 2 reports mean values of the Academic Self-efficacy factors separately for each year of enrollment in an undergraduate degree course. The three groups showed strengths in “Information Retrieval,” but weaknesses in “Working in Groups” and “Management of Relationships with Teachers.” Furthermore, the results of the univariate ANOVA and *post hoc* comparison based upon Tukey test showed a meaningful difference between first year students and second and third year students [*F*(df = 2, 751) = 9.46, *p* < 0.001, η^2^ = 0.024] in their levels of “Working in Groups.” Additionally, results showed a significant difference between first year students and second and third year students [*F*(df = 2, 751) = 3.79, *p* < 0.05, η^2^ = 0.010] in their levels of “Management of Relationships with Teachers.”

**FIGURE 3:**
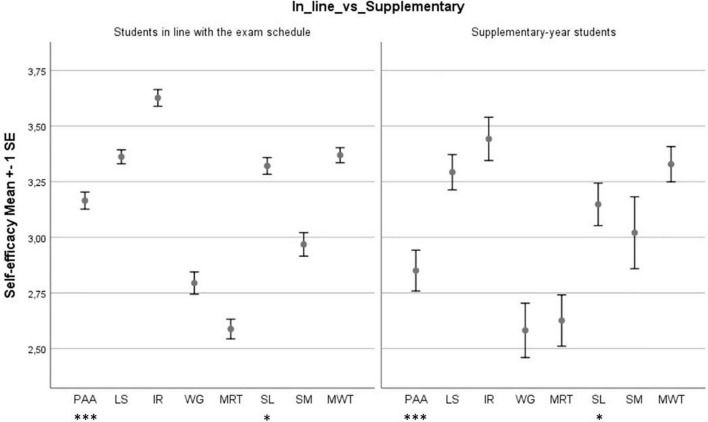
Mean levels of the Academic Self-efficacy factors for students in line with the exam schedule vs. supplementary-year students and *t*-test results. *p < 0.05; ***p < 0.001; PAA, “Planning Academic Activities”; LS, “Learning Strategies”; IR, “Information Retrieval”; WG, “Working in Groups”; MRT, “Management of Relationships with Teachers”; SL, “Skills for Lessons”; SM, “Stress Management”; MTW, “Management of Thesis Work.”

Finally, Figure 3 shows mean values of Academic Self-efficacy for students in line with the exam schedule vs. supplementary-year students. Both groups showed strengths in “Information Retrieval,” but weaknesses in “Management of Relationships with Teachers” and “Working in Groups.” Students in line with the exam schedule showed strengths in “Planning Academic Activities,” while supplementary-year students showed weakness in this factor. Furthermore, the results of the *t*-test showed a meaningful difference between students in line with the exam schedule and supplementary-year students in their levels of “Planning Academic Activities” [*t*(df = 827) = 3.63, *p* < 0.001, Cohen’s *d* = 0.43], and “Skills for Lessons” [*t*(df = 827) = 2.14, *p* < 0.05, Cohen’s *d* = 0.25].

## Discussion

The present study aimed to present the Academic Self-Efficacy Scale, a new multifaceted tool designed to measure self-efficacy beliefs in managing academic tasks among university students. The new scale presents adequate psychometric properties, the presence of measurement invariance, and associations with academic performance and experiences, and a remarkable discriminative validity. Analyses exploring the structure of the scale showed that it is made up of eight factors referring to the students’ perceived abilities to manage tasks and situations that are crucial for their successful academic path, namely planning activities to be done, implementing effective learning strategies during lessons and at home, retrieving information, working with peers, managing relationships with teachers, managing negative emotions and stress, and thesis work. All these factors are in line with the self-efficacy features found in the literature (Bandura, 1997; Cheung and Kwok, 1998; Amenkhienan and Kogan, 2004) and cover a wide variety of the efficacy beliefs related to the academic context. In particular, results showed the crucial role of “Planning Academic Activities,” related to the proportion of exams passed, the average exam rating for all the students independent of the enrollment year, the ability to manage stress, and with the ability to stay in line with academic achievement. Results are similar to the Life Design approach (Savickas et al., 2009), which underlined that the ability to plan personal aims and the next career steps is fundamental to career construction and career development. Findings from our study seem to suggest to focus on planning ability to develop intervention activities to support undergraduate students. In addition, our results showed that “Learning Strategies” and “Skills for Lessons” have relationships with the proportion of exams passed and the average exam rating for most of the students, even though they are not as strong as the ability to plan activities. In that sense, our results have highlighted that it is important to develop good study strategies and learn directly from class lectures, even though these are secondary to the ability to plan career steps. Finally, a strong negative correlation arose between “Working in Groups” and stress-related difficulties, showing how important it is to focus on the peer group to manage stress. In part, our study confirms findings present in the literature: self-efficacy assumes a key role in career planning and academic achievement. Our results showed that students with higher levels of self-efficacy are the ones who are in line with a traditional academic path and with academic goals. This particular result seems crucial to create specific interventions and promoting different levels of self-efficacy beliefs for different steps in the university career.

Differences between male and female students were found in their levels of “Planning Academic Activities,” “Information Retrieval,” and “Skills for Lessons”; these results confirm a female advantage in academic as stated by Eurostat data on European population and by previous researches (Ceci et al., 2014; Voyer and Voyer, 2014). Furthermore, our results showed higher levels of “Stress Management” in male students; these findings are in line with researches that show that female students are more likely to be influenced by academic stress and that perceived themselves are less able to manage it than male students (Ye et al., 2018). Surprisingly, our results showed higher levels of “Management of Relationships with Teachers” in male students; previous studies established closer and less conflictual relationships between teachers and girls than boys (Baker, 2006; Spilt et al., 2012). This result should be investigated further, considering also the possible influence due to teacher gender and its interaction with students’ gender. Moreover, the study confirms previous results (Bandura, 1997): students in line with the traditional path at the university have higher levels of self-efficacy, particularly in the ability to plan and use information from the classroom in a formative way. Even in this case, our study suggests that self-efficacy beliefs support personal competences and that they are fundamental to developing personal and professional skills, useful for the academic context, but also for future planning, as highlighted in previous research (Pajares and Urdan, 2005; Brausch, 2011; Azizli et al., 2015).

Despite its strengths, our study has some limitations. First, since this study was conducted on Italian university students and considering the possible variation among the different university systems, additional work is needed to confirm the generalizability of the scale to other cultural contexts. Activities and tasks required to the students, and following related self-efficacy beliefs, may be different if, as in the Italian system, there are no penalties after an exam failed several times compared to other university systems in which the maximum number of exam failures is limited. Considered possible differences among university systems, future research could explore the structure of the scale in different languages and other countries. Further, although we tested convergent validity by exploring the relationship between the academic self-efficacy and the academic experiences scales, future studies should explore convergent validity in more detail. This will not be easy since the available scales only focus on specific aspects on self-efficacy or are limited to specific disciplines. Yet, future research is needed on this aspect. Finally, even if the use of self-reported academic grades is widely accepted in the social sciences (Stone et al., 1999; Kuncel et al., 2005; Baumeister et al., 2007; Sticca et al., 2017), further studies could explore the role of self-efficacy on different outcomes. Other methods would be useful to assess the truthfulness of participants’ reported information, as data from university administrations concerning students’ performance indicators or a proxy assessment of self-efficacy. In this way, the amount of missingness in the variables collected would be less.

The development of the Academic Self-efficacy Scale could be a significant contribution to the literature and to intervention in vocational guidance. Measuring self-efficacy beliefs has important implications for school counselors, career counselors, and psychologists working in the academic field. The scale could be used on two levels: preventing academic failure or dropout, and helping struggling students. The results showed that the scale could be a good instrument to identify the students’ features and to intercept students with low levels of self-efficacy beliefs and those with more self-concern. The aim could be to create particular interventions for individuals, small groups (Koen et al., 2012) or large groups (Camussi et al., 2017), depending on the specific courses, the level of self-efficacy beliefs or their particular academic paths, to co-construct a new perception about their abilities. Moreover, the scale could be an instrument to build specific interventions and actions dedicated to sustaining the co-construction of academic motivation (Vallerand and Bissonnette, 1992; Vallerand et al., 1992; Vallerand, 2000) and general academic wellbeing. Starting from self-efficacy beliefs, the counselors could encourage the students to experiment with new strategies, promoting a new vision of their abilities, specifically in the new academic context, but expandable in the future working world. In that sense, the Academic Self-efficacy Scale could be a specific and brief instrument, helpful for working in synergy to implement a new representation of students and their abilities, to sustain academic and career success.

## Data Availability Statement

The datasets generated for this study are available on request to the corresponding author.

## Ethics Statement

The studies involving human participants were reviewed and approved by University of Milano-Bicocca. The patients/participants provided their written informed consent to participate in this study.

## Author Contributions

AG and PS contributed conception and design of the study. AG and CA organized the database and wrote the first draft of the manuscript. AG and NP performed the statistical analysis. NP, EC, GR, and PS wrote sections of the manuscript. All authors contributed to manuscript revision, read and approved the submitted version.

## Conflict of Interest

The authors declare that the research was conducted in the absence of any commercial or financial relationships that could be construed as a potential conflict of interest.

## Publisher’s Note

All claims expressed in this article are solely those of the authors and do not necessarily represent those of their affiliated organizations, or those of the publisher, the editors and the reviewers. Any product that may be evaluated in this article, or claim that may be made by its manufacturer, is not guaranteed or endorsed by the publisher.
