# Supplementary material for: Self-Efficacy Beliefs of University Students: Examining Factor Validity and Measurement Invariance of the New Academic Self-Efficacy Scale
Source: Front Psychol. 2022 Jan 13;12:498824. doi: 10.3389/fpsyg.2021.498824 (PMC8793353; doi:10.3389/fpsyg.2021.498824)
Supplement: Supplementary file 1 [file Data_Sheet_1.pdf]

Supplementary material. English and Italian version of the items of the University Academic Self-efficacy Scale

How well can you…	Quanto sei capace di…
. keep with the study schedule you set up	. rispettare sempre il programma di studio che hai stabilito
. sort what you have to study in the time you have left to prepare for an exam	. suddividere il materiale di studio, nel tempo che hai a disposizione per preparare un esame
. keep up continuous study habits throughout the school year	. mantenere un ritmo costante di studio lungo tutto l'anno accademico
. organize your time in order to finish a paper by the deadline	. organizzare il tempo a tua disposizione, per portare a termine un elaborato, rispettando tutte le scadenze
. plan the number of exams you will take in each session based on how difficult they are	. programmare il numero di esami da sostenere in ogni sessione, in base alla loro difficoltà
. set achievable goals by knowing your abilities and your limitations	. stabilire degli obiettivi raggiungibili, valutando le tue capacità e i tuoi limiti
. make connections, analogies and distinctions among the various subjects you are taking	. creare collegamenti e individuare analogie e differenze nei diversi materiali che stai studiando
. at the exam, convey in writing what you'd studied	. rielaborare adeguatamente in forma scritta, in sede d'esame, gli argomenti che hai studiato
. enhance your exam preparation with personalized, in-depth study	. arricchire la preparazione degli esami con approfondimenti personali
. adjust your way of expressing yourself according to the situation and the person you're talking to	. adeguare il tuo modo di relazionarti al contesto e all'interlocutore che hai davanti
. demonstrate your knowledge of that you've studied in an oral exam	. esporre adeguatamente in forma orale, in sede d'esame, gli argomenti che hai studiato
. focus on the main points of what you are studying	. concentrarti soprattutto sui concetti principali di una materia di studio
. get the information you need about administrative offices (opening times, how to contact them...)	. reperire le informazioni che ti sono necessarie riguardo le segreterie (orari d'apertura, modalità d'accesso…)
. get information from the university website	. reperire informazioni dal sito internet dell'università
. regularly check the departmental notice board to get information about your degree course	. raccogliere costantemente le informazioni riguardanti il tuo corso di studi, consultando le bacheche di facoltà
. get information on exam formats ahead of time	. raccogliere sempre in anticipo le informazioni riguardanti le modalità di svolgimento di un esame
. sign up for exams within the established timeline	. iscriverti sempre agli esami nei tempi stabiliti
. find out ahead of time if there are any prerequisite exams to take in your degree course before beginning other courses	. informarti, sempre in anticipo, sulla presenza di propedeuticità nel tuo corso di laurea
. start efficient study groups	. promuovere la creazione di efficienti gruppi di studio
. use good group study strategies (quiz each other, etc.)	. utilizzare adeguate strategie di studio in gruppo (ad es. interrogarsi a vicenda, ecc.)
. work together productively by defining specific goals and tasks	. lavorare in gruppo in modo produttivo, definendo obiettivi e compiti precisi
. raise your hand to ask the professor to explain parts of the lesson that you don't understand	. intervenire in aula per chiedere chiarimenti al docente, in merito a contenuti della lezione che non hai compreso
. participate actively in in-class discussion	. partecipare attivamente alla discussione d'aula
. go to your professors to get useful information on courses	. far riferimento ai tuoi docenti al fine di reperire informazioni utili sui corsi
. stay focused in class even when is is noisy or crowded	. mantenere alta la concentrazione durante la lezione, anche quando in aula si verificano situazioni di disturbo (rumori, aule affollate, guasti…)
. attend class regularly even when the exam session approaches	. frequentare con costanza le lezioni, anche quando si avvicina la sessione d'esame

. take clear, useful notes in class<br><br>. glean and reprocess the essential points in a lecture	. prendere appunti chiari ed utili, durante la lezione<br><br>. cogliere e rielaborare i contenuti essenziali di una lezione
. keep exam anxiety under control<br><br>. avoid getting discouraged when you fail an exam	. controllare adeguatamente l’ansia che sorge nel contesto d’esame<br>. non scoraggiarti in seguito ad un esame fallito

Supplementary material. English and Italian version of the items of the University Academic Self-efficacy Scale Related to Thesis Work

**How well can you…**	**Quanto sei capace di…**
. select what is useful from all your research to write your thesis	. selezionare tra tutto il materiale reperito, quello che è effettivamente utile per il tuo elaborato finale
. use a clear and coherent structure to organize your research material for the thesis	. organizzare il materiale raccolto per l'elaborato finale secondo una struttura chiara e coerente
. devise a good project for your thesis	. ideare un buon progetto per il tuo elaborato finale
. make good use of your advisor's suggestions to write your thesis	. utilizzare al meglio i consigli e le indicazioni che ti vengono suggerite dal relatore, per la stesura dell'elaborato finale
. work continually in order to finish your thesis in time	. lavorare costantemente, in modo da completare l'elaborato finale nei tempi stabiliti
. use library resources to find materials for your thesis	. reperire il materiale per l'elaborato finale, utilizzando le risorse presenti in biblioteca
. respect all graduation deadlines (getting a thesis advisor, graduation application, handing in documents...)	. rispettare tutte le scadenze relative al conseguimento della laurea (attribuzione relatore, domanda di laurea, consegna documenti…)
